# Acute Myeloid Leukemia (AML) With T-Cell Differentiation Arising From Chronic Myelomonocytic Leukemia (CMML)

**DOI:** 10.1155/crh/5584297

**Published:** 2024-12-14

**Authors:** Anthony Crymes, Mark G. Evans, Deepa Jeyakumar, Jerry J. Lou, Xiaohui Zhao, Sherif A. Rezk

**Affiliations:** ^1^Department of Medicine, Keck School of Medicine, University of Southern California, Los Angeles, California, USA; ^2^Department of Pathology, Caris Life Sciences, Phoenix, Arizona, USA; ^3^Department of Medicine, Hematology and Oncology Division, University of California Irvine (UCI) Medical Center, Orange, California, USA; ^4^Department of Pathology and Laboratory Medicine, University of California Irvine (UCI) Medical Center, Orange, USA

**Keywords:** acute myeloid leukemia, chronic myelomonocytic leukemia, T-cell differentiation

## Abstract

Chronic myelomonocytic leukemia (CMML) is a myelodysplastic/myeloproliferative neoplasm characterized by peripheral blood monocytosis and bone marrow dysplasia. In approximately one-fourth of cases, CMML can demonstrate progression to acute myeloid leukemia (AML), referred to as AML ex CMML. We present a 58-year-old woman with a past medical history of idiopathic thrombocytopenic purpura (ITP) who demonstrated 24% bone marrow blasts on a repeat biopsy obtained two years after being diagnosed with CMML. By the flow cytometric analysis, the blasts expressed partial CD34, CD13, CD117, partial MPO, and partial CD123 with coexpression of the T-lymphoid markers CD2, CD5, CD7, partial CD4, cytoplasmic CD3, partial cytoplasmic TDT, and CD38, suggestive of AML with rare mixed myeloid/T-cell phenotype. Treatment with various agents including decitabine, cytarabine, daunorubicin, etoposide, and venetoclax, and two experimental bromodomain and extraterminal (BET) inhibitors did not produce sustained remissions, and the patient eventually succumbed to her disease. T-cell phenotype is an exceedingly rare feature of AML ex CMML, and whether this unique differentiation pathway contributed to the aggressive disease course remains unclear.

**Trial Registration:** ClinicalTrials.gov identifier: NCT02543879, NCT03360006

## 1. Introduction

Chronic myelomonocytic leukemia (CMML) is a hematopoietic malignancy defined as peripheral blood monocytosis > 1 × 10^9^/L and dysplasia involving at least one myeloid lineage, with other myeloproliferative neoplasms having been excluded in the World Health Organization (WHO) classification [1]. Further subcategories (CMML-1 and CMML-2) have been designated according to the number of peripheral blood and bone marrow blasts, which are reported to be an important prognostic indicator [2, 3].

CMML is generally rare, with an annual incidence estimated at 0.4 cases per 100,000 population, and progresses to acute myeloid leukemia (AML) in approximately 20%–30% of cases [4, 5]. The WHO describes this progression as AML with myelodysplasia-related changes—a subtype that is often aggressive and includes a heterogeneous group of acute leukemias that either arise from a previous myelodysplastic syndrome or myelodysplastic syndrome/myeloproliferative neoplasm, demonstrating a myelodysplastic syndrome-related cytogenetic abnormality or displaying significant multilineage dysplasia [1]. AML with myelodysplasia-related changes arising in a background of CMML has been termed in one study as “AML ex CMML” [6]. Typically, the few reported cases of this entity demonstrate clear myeloid and monocytic differentiation, but none have expressed T-cell markers as seen in our recent patient to the best of our knowledge.

## 2. Case Report

A 58-year-old Caucasian woman presented to her primary care physician with a large thigh bruise and was diagnosed with idiopathic thrombocytopenic purpura (ITP). She was treated with steroids, intravenous immunoglobulins, and rituximab without considerable improvement, and eventually underwent splenectomy. Her complete blood count (CBC) was closely monitored for three years, at which time she developed persistent monocytosis and underwent bone marrow biopsy at an outside hospital, which reportedly showed 80%–90% cellularity with multilineage dysplasia and no apparent increase in blasts. Flow cytometry confirmed < 5% CD34/CD117-positive blasts, but approximately 20% monocytes. Cytogenetic studies revealed a normal female karyotype. These findings were consistent with CMML-1.

**Figure 1:**
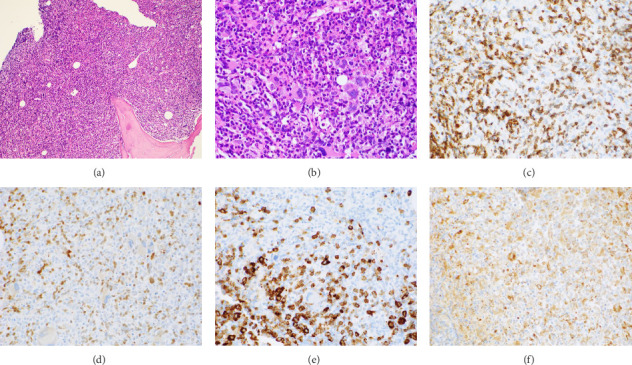
Hematoxylin and eosin staining of the patient's hypercellular bone marrow, featuring dysmegakaryopoiesis and an increased proportion of immature blasts with fine nuclear chromatin, 100x (a) and 200x (b) magnification. By immunohistochemistry, the blasts were positive for CD2 (c) and CD5 (d), with partial expression of MPO (e), 200x magnification. Strong CD68 (f) staining is suggestive of increased monocytic proliferation, 200x magnification.

The patient opted for watchful waiting, and no therapies aimed at treating the CMML, including stem cell transplant. Two years later, she experienced several months of progressive weakness, and her CBC was notable for a white blood cell count of 78.5 thousand per microliter, which included 19% blasts (18.9 thousand per microliter). Repeat bone marrow biopsy demonstrated 24% blasts that expressed partial CD34, CD13, CD117, partial MPO, and partial CD123 with coexpression of the T-lymphoid markers CD2, CD5, CD7, partial CD4, cytoplasmic CD3, partial cytoplasmic TDT, and CD38, as shown in Figure 1. The cytogenetic analysis was notable for trisomy 21. The patient was initially treated with a 10-day course of decitabine, followed by 7 + 3 induction chemotherapy with cytarabine and daunorubicin, and later five doses of decitabine. Repeat bone marrow biopsy confirmed persistent disease at Day 28, and the patient then completed eight cycles of the experimental bromodomain and extraterminal (BET) inhibitor FT-1101 on clinical trial. She subsequently received three doses of etoposide and cytarabine and was later transitioned to four cycles of venetoclax and decitabine. Rising blast count prompted the patient to enroll in a Phase 1 clinical trial of ABBV-744. She received four doses of this BET inhibitor before suffering a ground-level fall, which resulted in a left psoas muscle hematoma. The patient was hospitalized for lumbar artery embolization, but she developed acute respiratory failure and was placed on hospice care, expiring shortly thereafter. A complete and unrestricted autopsy attributed the cause of death to pulmonary congestion and edema, exacerbated by the aggressive diuresis used in the treatment of tumor lysis.

## 3. Discussion

Our patient presented with acute leukemia secondary to CMML, expressing a rare mixed myeloid/T-cell phenotype. Courville et al. published the largest series of 38 patients with so-called “AML ex CMML,” none of which expressed T-cell phenotype on the leukemic blasts [6]. The authors observed an approximate 2:1 male to female ratio in instances of AML ex CMML. In contrast to de novo AML, patients with myeloid leukemia secondary to CMML were significantly older (median 59 versus 71 years, respectively) and had considerably poorer survival (17 versus 6 months, respectively). In the study, AML ex CMML compared to other cases of AML with myelodysplasia-related changes showed similarly poor prognosis and no significant difference in gender association or age of onset.

De novo AML and AML with myelodysplasia-related change cases were much less frequently diagnosed as M4 or M5 according to the original French American British (FAB) classification system than cases with AML ex CMML, which usually exhibit a clear myeloid and/or monocytic differentiation. In our case, however, a mixed myeloid/T-cell phenotype was noted for the AML ex CMML. The leukemic blasts expressed CD34 along with the myeloid markers CD13, partial MPO, and CD117, and the T-cell markers cytoplasmic CD3, partial cytoplasmic TdT, CD2, CD5, CD7, and partial CD4. AML with T-cell differentiation has been well documented, and it typically manifests later in life (median age 63 years) and affects men twice more frequently than women [7]. A more aggressive clinical course and poor survival rates are typically reported for these cases, averaging approximately 8 months, compared to classical AML (17 months).

Our case followed a clinical course that might be expected according to the medical literature for patients with AML ex CMML. Her CMML was initially diagnosed at age 61 on a bone marrow biopsy with no increased proportion of blasts. And 2 years later, a repeat biopsy demonstrated acute leukemia in a background of persistent multilineage dysplasia and peripheral blood monocytosis, suggestive of transformation as opposed to a de novo process. No molecular alterations were reported that could have been utilized for targeted therapy, and the patient passed away 10 months after enrolling in two clinical trials. Ultimately, our case is novel in that, while most instances of AML transforming from CMML demonstrate clear myeloid and monocytic markers, this patient's neoplasm is the first to our knowledge that shows evidence of T-cell differentiation. Moreover, her clinical history should alert treating physicians that AML ex CMML with T-cell phenotype may be associated with aggressive disease behavior, resulting in more likely treatment failures and shorter overall survival. In particular, the patient's unique leukemia never achieved a sustained remission despite multiple lines of therapy, including experimental BET inhibitors. One might also advocate for the use of advanced molecular diagnostics, including next-generation sequencing (NGS) that was not readily available at the time this patient was receiving care, when attempting to treat a similar AML with an unusual phenotype. NGS might possibly have revealed additional therapeutic options. In the future, it may be important for clinicians treating AML ex CMML to carefully consider the blastic phenotype when selecting the appropriate diagnostic tests and treatments for their patients.

## Data Availability

The data that support the findings of this study are available from the corresponding author on request. The data are not publicly available due to privacy or ethical restrictions.
